# The Effect of Phosphorylation on the Electron Capture Dissociation of Peptide Ions

**DOI:** 10.1016/j.jasms.2008.05.015

**Published:** 2008-09

**Authors:** Andrew J. Creese, Helen J. Cooper

**Affiliations:** School of Biosciences, University of Birmingham, UK Birmingham, United Kingdom

## Abstract

The effect of site and frequency of phosphorylation on the electron capture dissociation of peptide ions has been investigated. The ECD of a suite of synthetic peptides (APLSFRGSLPKSYVK; one unmodified, three singly-phosphorylated, three-doubly phosphorylated, and one triply-phosphorylated); two tryptic phosphopeptides (YKVPQLEIVPN_p_SAEER, α-casein and FQ_p_SEEQQQTEDELQDK, β-casein) and their unmodified counterparts, were determined over a range of ECD cathode potentials. The results show that, for doubly-charged precursor ions, the presence of phosphorylation has a deleterious effect on ECD sequence coverage. The fragmentation patterns observed suggest that for peptides with multiple basic residues, the phospho-groups exist in their deprotonated form and form salt-bridges with protonated amino acid side chains. The fragmentation observed for the acidic tryptic peptides suggested the presence of noncovalent interactions, which were perturbed on phosphorylation. Increasing the ECD electron energy significantly improves sequence coverage. Alternatively, improved sequence coverage can be achieved by performing ECD on triply-charged precursor ions. The findings are important for the understanding of gas-phase fragmentation of phosphopeptides.

## Graphical Abstract

Phosphorylation has a deleterious effect on the ECD of doubly-charged basic peptides due to salt bridge formation between deprotonated phospho-groups and protonated lysine/arginine side chains.

**Figure:**
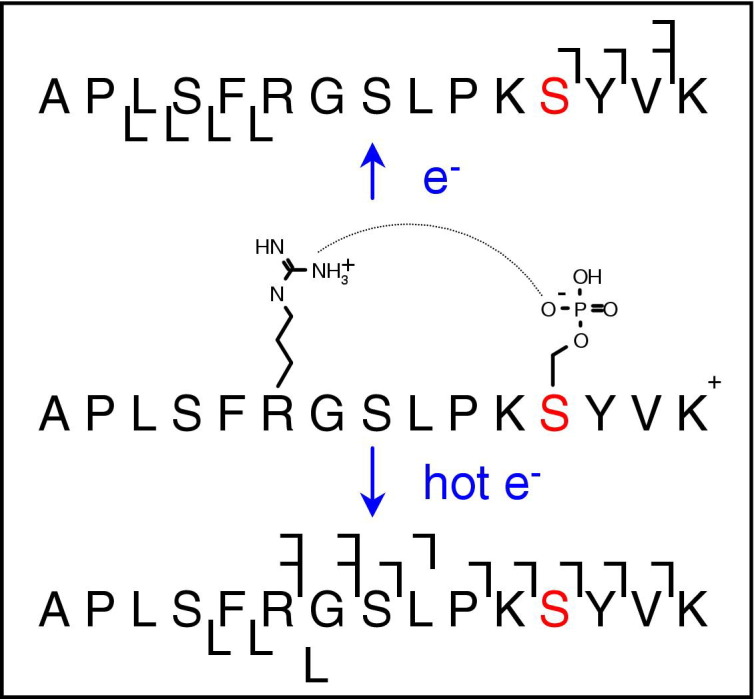

The introduction of electron capture dissociation (ECD) [1] in 1998 provided a unique fragmentation technique for biomolecular analysis. ECD is primarily employed in Fourier transform ion cyclotron resonance (FT-ICR) mass spectrometers. (It is possible to perform ECD in an ion trap [2, 3, 4] but fragmentation efficiency is not yet comparable to FT-ICR). In ECD, ions of interest are irradiated with low-energy electrons (≈0.2 eV) producing charge-reduced radical ions which dissociate via radical driven pathways [5, 6]. ECD differs from classic tandem mass spectrometry (MS/MS) techniques such as collision induced dissociation (CID) [7, 8] or infrared multi-photon dissociation (IRMPD) [9, 10]: ECD is believed to be nonergodic, whereas CID and IRMPD are thermal methods in which the lowest energy bonds are cleaved first [11]. In ECD, peptide backbone cleavage occurs at N–Cα bonds leading to *c* and *z*· (or *c*· and *z* [12, 13] fragments. In CID and IRMPD, cleavage of N–CO bonds to produce *b* and *y* fragments [14] is observed. There are significant advantages gained by fragmentation of peptides with ECD: the apparent nonergodic nature of ECD means it is random and relatively nonselective, the only exception being cleavage N-terminal to proline [15]. The nonselective nature of ECD means the extent of peptide sequence coverage is greater than for CID [16, 17]. For peptides with labile post-translational modifications (PTMs), loss of the PTM is usually the dominant fragment observed in CID and IRMPD. In ECD, PTMs are retained on the peptide backbone [18]. The site of modification can therefore be localized; γ-carboxyglutamic acid [18], phosphorylation (S, T and Y) [19, 20], N- and O- glycosylation [21, 22], acylation [23], and sulfation [18] are examples of modifications localized by ECD.

The application of ECD to phosphorylation analysis is of great interest. It is estimated that up to one-third of all proteins in eukaryotes are phosphorylated [24]. Phosphorylation and dephosphorylation control complex processes such as signal transduction [25], cell cycle, cell growth, and metabolism. They also modulate protein activity, stability, interaction, and localization [26, 27]. The advent of ECD has provided a method for accurately identifying the sites of modification in phosphopeptides/proteins [19, 20]. CID of phosphopeptides tends to result in the loss of phosphoric acid H_3_PO_4_ (98 Da) from phospho-serine/threonine and (less so) the phosphate group HPO_3_ (80 Da) from phosphotyrosine [28] at the expense of sequence fragments. The loss 98 or 80 Da confirms the presence of phosphorylation; however, in the absence of sequence fragments, the site can only be located if there is one possible modification site (S, T, Y) and the sequence of the peptide is known. Because ECD offers greater peptide coverage and phosphorylation is retained on backbone fragments, the probability of localization is increased [29]. To date ECD has been applied to the localization of sites of phosphorylation in the proteins human α-casein [30], cardiac troponin I [31], and Sprouty2 [32], thereby demonstrating its potential for solving “real” biological problems.

The introduction of data dependent on-line liquid chromatography (LC)-ECD MS/MS has allowed automated analysis of complex protein samples. We have recently demonstrated data dependent nano-LC ECD MS/MS for the identification of the protein ROR2 isolated from human chondrocytes [33]. Because ECD and CID produce different backbone fragments, they are complimentary methods for peptide identification and combined provide a large amount of information. Zubarev and coworkers have developed a method performing both ECD and CID on all eluting peptides [34, 35, 36, 37, 38]. This combined approach provides information about the relationship between *b/y* and *c/z*· fragments, i.e.; the “golden rules” [39]. Recent studies in our laboratory have shown that on-line LC-ECD MS/MS identifies peptides with greater confidence than on-line LC-CID MS/MS in terms of the extent of sequence tag [17]. Nevertheless, the time scale for ECD remains considerably longer than that for CID. The result is that fewer peptides are selected for fragmentation in an LC-ECD MS/MS analysis. This is particularly pertinent in the case of phosphopeptide analysis; phosphopeptides tend to exist at lower stoichiometry than their unmodified counterparts and can be overlooked. Enrichment of protein digests for phosphopeptides by use of titanium dioxide [40] or zirconium dioxide [41] affinity chromatography alleviates the problem to some extent. We have developed two targeted LC-ECD approaches that focus ECD time on phosphopeptides. In neutral loss (NL) ECD [42], protein digests are separated by on-line LC. All eluting peptides are subjected to CID. If a neutral loss corresponding to loss of phosphoric acid (−98 Da) is observed in the CID mass spectrum, ECD of the precursor ion is triggered. In targeted ECD [32], the protein digest is first analyzed by LC-CID MS/MS and putative phosphopeptides identified by mass. An LC-ECD MS/MS analysis is then performed in which only potential phosphopeptides are investigated. These methods combine the high scan rate of CID with the improved peptide sequence coverage and the retention of PTMs of ECD.

Here, we investigate the effect of site and frequency of peptide phosphorylation on electron capture dissociation behavior. We have compared the ECD of eight synthetic phosphopeptides (APLSFRGSLPKSYVK) (one unmodified, three singly-phosphorylated, three-doubly phosphorylated, and one triply-phosphorylated) over a range of ECD cathode potentials. We have also investigated the behavior of two tryptic phosphopeptides, one from α-S1-casein (YKVPQLEIVPN_p_SAEER) and one from β-casein (FQ_p_SEEQQQTEDELQDK), and their nonphosphorylated counterparts. The results show that for doubly-charged peptide precursor ions the presence of phosphorylation has a deleterious effect on ECD sequence coverage. The fragmentation pattern observed suggests this is the result of the deprotonated phospho-group forming salt bridges with protonated side chains of basic amino acid residues. Increasing the ECD electron energy (by decreasing the ECD cathode potential) significantly improves sequence coverage. It is postulated that the deposition of additional energy facilitates cleavage of the noncovalent bonds enabling detection of the backbone fragments, in a similar manner to activated ion ECD [43]. Improved sequence coverage can also be obtained by ECD of triply-charged precursor ions. The tryptic peptides showed improved sequence coverage with increasing electron energy for both phosphopeptides and their unmodified counterparts. These results suggest that for these peptides the phospho-group was not directly involved in noncovalent bonding but that its presence altered the peptide conformation and intramolecular bonding.

## Experimental

### Preparation of Synthetic Peptides

A suite of eight synthetic peptides (APLSFRGSLPKSYVK: one unmodified, three singly-phosphorylated, three doubly-phosphorylated, and one triply-phosphorylated) were synthesized by Alta Biosciences (Birmingham, UK) and used without further purification. The peptides were diluted to 2 pmol/μL in methanol (Fisher Scientific, Leicestershire, UK):water (J. T. Baker, Deventer, The Netherlands) (75:25) + 1% formic acid (Fisher Scientific). α-Casein and β-casein (Sigma Aldrich, Gillingham, UK) were digested with trypsin (Sigma Aldrich) (1:50, enzyme to protein, wt/wt) in 50 mM ammonium bicarbonate (Sigma Aldrich) (pH 8) at 37 °C overnight. The protein digests were dephosphorylated with calf intestinal alkaline phosphatase (1 unit per μg protein) (New England Biolabs, Ipswitch, MA) at pH 8 for 1 h at 37 °C. The digests were diluted to a final concentration of ∼2 pmol/μL in methanol:water (75:25) + 1% formic acid.

### ECD-MS/MS

All tandem mass spectrometry analysis was performed on a Thermo Finnegan LTQ FT mass spectrometer (Thermo Fisher Scientific, Bremen, Germany). Samples were injected by use of an Advion Biosciences Triversa electrospray source (Advion Biosciences, Ithaca, NY) at a flow rate of 200 nL/min.

The mass spectrometer alternated between a full FT-MS survey scan (*m/z* 400–1800) and 32 subsequent ECD-MS/MS scans of the peptide precursor ions with decreasing cathode potential, from −1.34 to −16.34 V (0.5 V stepwise) and one ECD scan at cathode potential of 0 V. Survey scans were acquired in the ICR cell with a resolution of 50,000 at *m/z* 400. Precursor ions were isolated in the ion trap and transferred to the ICR cell for ECD analysis. The isolation width was 7.5 Th. Automatic gain control was used to accumulate precursor ions in the ion trap (target 5 × 10^5^, maximum fill time 2 s). Electrons for ECD were produced by an indirectly heated barium-tungsten cylindrical dispenser cathode (5.1 mm diameter, 154 mm from the cell, 1 mm off axis) (Heat-Wave Labs, Watsonville, CA). The current across the electrode was 1.1 A. Ions were irradiated with electrons for 70 ms. Each ECD scan consisted of 3 co-added microscans acquired with resolution of 25,000 at *m/z* 400.

Data were analyzed using Xcalibur 2.05 software (Thermo Fisher Scientific). All mass spectra were manually searched for *a, b, c*/*c*·*, y*, and *z*·/*z* fragment ions.

## Results and Discussion

**Figure 1:**
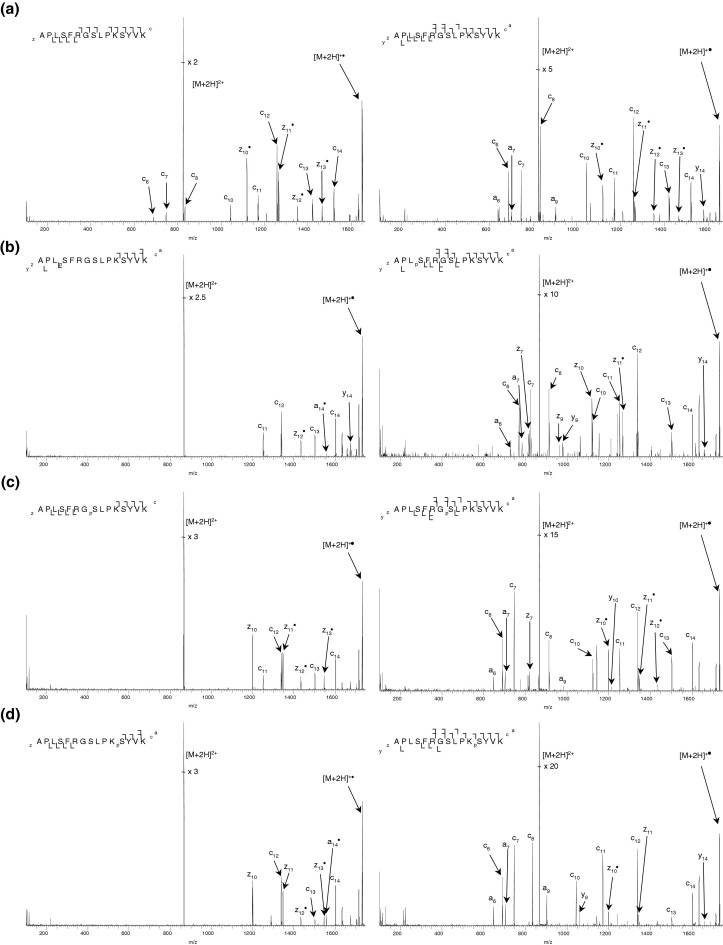
ECD mass spectra of doubly-charged ions of four synthetic peptides (APLS1FRGS2LPKS3YVK). ECD of (**a**) unmodified peptide at cathode potentials of (left) −3.34 V (standard) and (right) −13.34 V (cathode potential which gave the greatest sequence coverage) (6 scans); (**b**) Ser1 phosphopeptide at cathode potentials of (left) −3.34 V and (right) 14.34 V (4 scans); (**c**) Ser2 phosphopeptide at cathode potentials of (left) −3.34 V and (right) −14.34 V (7 scans); (**d**) Ser3 phosphopeptide at cathode potentials of (left) −3.34 V and (right) −14.84 V (9 scans).

**Figure 2:**
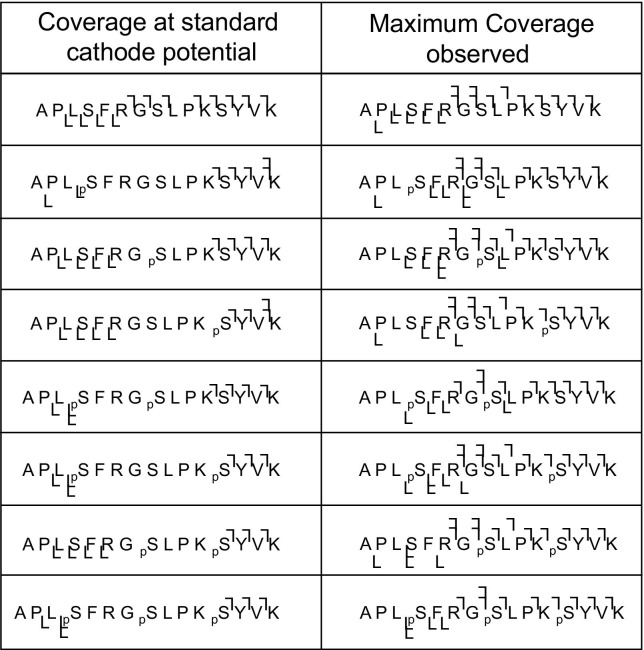
Summary of fragments observed following ECD of doubly-charged precursor ions at ‘standard’ cathode potential and at the cathode potential which gave greatest sequence coverage.

Figure 1 shows the ECD mass spectra of each of the doubly-charged ions of the unmodified and three singly-phosphorylated synthetic peptides at the “standard” cathode potential (−3.34 V), i.e.; the cathode potential applied in our LC-ECD MS/MS proteomics experiments, and at the cathode potential that gives the greatest peptide coverage, i.e., the greatest number of N–Cα cleavages. The ECD mass spectra of the doubly- and triply-phosphorylated peptides are shown in Supplementary Figure 1, which can be found in the electronic version of this article. Peptide sequence coverage is summarized in Figure 2. For all phosphopeptides, the maximum sequence coverage was obtained at ECD cathode potentials between −14 V and −15 V. After this point, higher mass fragments were not observed, in agreement with the findings of Tsybin et al. [44]. The ECD mass spectrum obtained for the unmodified peptide under standard conditions (Figure 1a, left) shows 12 of 14 N–Cα bonds cleaved, the maximum expected due to the two proline residues present. Additional sequence information is obtained at a cathode potential of −13.34 V (Figure 1a, right) with the mass spectrum revealing the presence of the *y*_14_ fragment resulting from cleavage N-terminal to Pro2. Presumably the additional electron energy has enabled this facile cleavage. The ECD mass spectra obtained from the doubly-charged singly-phosphorylated peptides are shown in Figure 1b, c, and d. For the peptide (APL_p_SFRGSLPKSYVK) (Figure 1b, left), five N-Cα bonds are cleaved following ECD at the standard cathode potential. The *y*_14_ fragment is also observed. The site of phosphorylation cannot be identified from this mass spectrum. The sequence coverage seen is significantly worse than that obtained for the unmodified peptide at the same cathode potential: there are no *z* fragments smaller than *z*_12,_ and no *c* fragments smaller than *c*_11_. The greatest coverage was achieved with an ECD cathode potential of −14.34 V (Figure 1b, right), 10 of the 14 bonds N–Cα were cleaved and the site of phosphorylation can be localized. Similar results were obtained for peptide APLSFRG_p_SLPKSYVK (Figure 1c). At the standard ECD cathode potential eight N–Cα bonds were cleaved. The site of phosphorylation can be identified by elimination, however no fragments adjacent to the site of modification were observed. No *c* ions smaller than *c*_11_ and no *z* ions smaller than *z*_10_ were observed. An ECD cathode potential of −14.34 V (Figure 1c, right) gave the greatest peptide coverage with 11 out of 14 N–Cα bonds cleaved. Fragments were observed adjacent to the site of phosphorylation. For peptide APLSFRGSLPK_p_SYVK (Figure 1d), 7 of the 14 N–Cα bonds were cleaved at the standard cathode potential. No *c* fragment ions smaller than *c*_12_ and no *z* ions smaller than *z*_10_ were identified. The site of phosphorylation could not be identified from this spectrum. The best coverage was achieved with an ECD cathode potential of −14.84 V (Figure 1d, right): 10 out of 14 N–Cα bonds were cleaved, in addition to production of the *a*_9_· and *y*_14_ fragments. The site of phosphorylation can be identified.

The ECD mass spectra of the multiply-phosphorylated peptides show the same trend as those of the singly-phosphorylated peptides (see Figure 2). The ECD mass spectrum of the doubly phosphorylated peptide (APL_p_SFRG_p_SLPKSYVK) (Supplementary Figure 1) shows cleavage of 6 of 14 N–Cα bonds. The sites of phosphorylation may be identified by elimination but there are no *c* fragments smaller than *c*_11_, and no *z* fragments smaller than *z*_12_·. Eleven out of fourteen backbone bonds were cleaved when the cathode potential is increased to −14.84 V. Ten of these result in *c* and *z*· ions and one of the cleavages produces a *y* ion. Note that the phosphorylation is retained on *y*_12_. The ECD mass spectrum of the peptide APL_p_SFRGSLPK_p_SYVK (Supplementary Figure 1B) obtained with standard cathode potential shows cleavage of 5 out of 14 N–Cα bonds, plus the *y*_12_ fragment ion. The sites of phosphorylation cannot be localized. The greatest coverage was achieved with an ECD cathode potential of −14.84 V (Supplementary Figure 1B, right): 10 of 14 N–Cα bonds were cleaved in addition to production of three *y* and three *a*· fragment ions. Both phosphorylation sites are identified. The ECD mass spectrum of the doubly-phosphorylated peptide APLSFRG_p_SLPK_p_SYVK (Supplementary Figure 1C) shows 7 of 14 N–Cα bonds were cleaved following ECD with standard cathode potential. The sites of phosphorylation may be identified by elimination; however, no fragments adjacent to the modification sites were observed. The greatest sequence coverage is achieved with an ECD cathode potential of −14.84 V: 9 of the 14 N–Cα bonds are cleaved. Both sites of modification are unambiguously identified. The ECD mass spectrum of the triply-phosphorylated peptide APL_p_SFRG_p_SLPK_p_SYVK (Supplementary Figure 1D) obtained with a standard cathode potential reveals cleavage of five of the 14 N–Cα bonds. The greatest coverage was achieved with an ECD cathode potential of −14.34 V. Eleven of the 14 N–Cα bonds were cleaved.

The results obtained for the doubly-charged singly-phosphorylated peptides can be explained by consideration of the charging pattern and the presence of noncovalent interactions in the form of salt bridges. In the unmodified peptide ion, the charge arises through protonation of the arginine residue and the C-terminal lysine. The arginine is the most basic amino acid residue and the C-terminal lysine the farthest site from the arginine. Electron capture occurs at the least basic site [45], i.e., the lysine. This is confirmed by the fragmentation pattern (Figure 1a) observed for the unmodified peptide: All the observed fragments contain the (protonated) arginine residue. The fragmentation pattern observed for doubly-charged APL_p_SFRGSLPKSYVK suggests that the phosphorylation is deprotonated and that the overall 2+ charge is achieved through protonation of the arginine and both lysines. Electron capture appears to occur at the C-terminal lysine and only phosphorylated fragments which contain both the arginine and the central lysine are observed (Figure 1b). However, the results cannot be explained by the charging pattern alone. For example, one might expect to observe fragments *z*_5_ and *z*_7_ through *z*_9_ if the central lysine was protonated. Moreover, doubly-charged *z*_10_ and *z*_11_ fragments might be observed and this was not the case. (Note, however, that it is likely any doubly-charged fragments and their negatively charged complements would be held together coulombically). It is postulated that the negatively-charged phosphate group forms a strong interaction—a salt bridge—with the positively charged lysine side chain [46]. It is well established that ECD tends not to cleave noncovalent interactions [43]. Any fragments from the sequence region between the phosphate group and the lysine would not be observed because, although the peptide backbone has dissociated, the fragments are held together by the salt bridge. This suggestion is further corroborated by the results obtained for doubly-charged APLSFRG_p_SLPKSYVK (Figure 1c). The fragmentation pattern suggests that the phosphorylation is deprotonated, both lysines and the arginine are protonated, and that salt bridge(s) exist between the phospho group and the protonated amino acid side chains. Electron capture appears to occur at the C-terminal lysine. Again only phosphorylated fragments which contain arginine and the central lysine are observed. This explanation accounts for the observation of *z*_10_ and *z*_11_· following ECD of [APLSFRG_p_SLPKSYVK]^2+^ but not following ECD of [APL_p_SFRGSLPKSYVK]^2+^. The ECD of doubly-charged APLSFRGSLPK_p_SYVK provides further confirmation—the *c*_11_ fragment is not observed (Figure 1d).

The situation is more complicated for the doubly-phosphorylated peptides (Supplementary Figure 1). If both phospho-groups are deprotonated, the overall charge derives from protonation of both lysines, the arginine and the N-terminus. The observed fragmentation patterns confirm this. Only phosphorylated fragments that contain arginine, central lysine, and either the N-terminus or C-terminal lysine are observed. For example, *c*_11_ is observed for APL_p_SFRG_p_SLPKSYVK, but not for APL_p_SFRGSLPK_p_SYVK or APLSFRG_p_SLPK_p_SYVK. Fragments *z*_10_· and *z*_11_· were observed for APLSFRG_p_SLPK_p_SYVK but not for either APL_p_SFRG_p_SLPKSYVK or APL_p_SFRGSLPK_p_SYVK. The fragmentation patterns suggest that multiple noncovalent interactions between deprotonated phospho groups and protonated amino acid side chains exist. Extension of this hypothesis to the triply-phosphorylated peptide suggests that all phosphorylations are deprotonated and the N-terminus, both lysines, the arginine, and another backbone amide nitrogen are protonated. Again, the observed fragmentation pattern suggests that the phospho-groups are deprotonated and that multiple salt bridges with protonated amino acid side chains exist.

**Figure 3:**
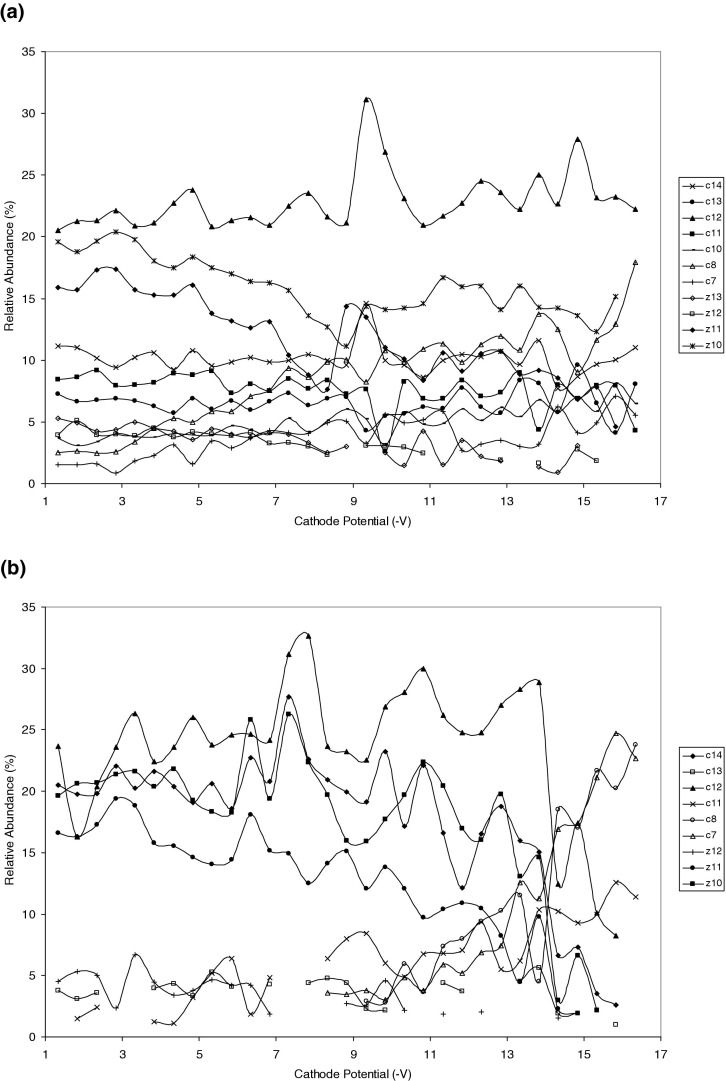
Normalized relative abundance of peptide fragment ions versus ECD cathode potential for (**a**) the unmodified peptide (APLSFRGSLPKSYVK); (**b**) the singly modified phosphopeptide (APLSFRGSLPK_P_SYVK).

Increasing the ECD electron energy by decreasing the ECD cathode potential resulted in greater sequence coverage for all of the phosphopeptides. These results suggest that deposition of greater amounts of energy on electron capture facilitates cleavage of the noncovalent bonds and can promote hydrogen rearrangement within the peptide, i.e.; reprotonation of the phospho-groups. In addition, cleavage of the noncovalent bonds increases conformational heterogeneity, which plays a role in increased peptide sequence coverage [47, 48]. For peptide APL_p_SFRGSLPKSYVK, increasing the ECD electron energy resulted in the appearance of fragments *z*_7_, *z*_9_, *z*_10_, *z*_11_·, and *c*_6_, *c*_7_, *c*_8_, *c*_10_. The appearance of *z_7_* and *z_9_* can be explained purely on the basis of cleavage of the salt bridge. Formation of the remaining fragments, however, must involve hydrogen rearrangement. Without that, *z*_10_, and *z*_11_· would be doubly-charged and *c*_6_, *c*_7_, *c*_8_, *c*_10_ would be neutral. Similarly, for peptide APSFRG_p_SLPKSYVK, the appearance of *z*_7_ and *c*_6_, *c*_7_ can be explained by straightforward cleavage of the salt bridge, however *c*_8_ and *c*_10_ require reprotonation of the phospho-group. Figure 3 shows the normalized relative abundance of the major ECD fragments from doubly-charged precursors of unmodified APLSFRGSLPKSYVK (Figure 3a) and singly-phosphorylated APLSFRGSLPK_p_SYVK (Figure 3b). Only fragments detected in four or more successive scans are included. The relative abundance was calculated as the ratio of the intensity of the fragment to the sum of the intensities of all the fragments (excluding the charge-reduced species). The unmodified peptide (APLSFRGSLPKSYVK) (Figure 3a) did not show marked differences in the normalized relative abundances of the fragments: Fragments *c*_11_, *z*_13_·, *z*_12_·, *z*_11_·, and *z*_10_· showed slight decreases, fragments *c*_12_, *c*_10_, *c*_8_, and *c*_7_ showed slight increases and fragments *c*_14_ and *c*_13_ did not vary with decreasing ECD cathode potential (increasing electron energy). Tsybin et al. [49] have shown previously that increasing electron energy correlates with a reduction in average fragment ion mass. In the case of the singly-phosphorylated peptide (APLSFRGSLPK_p_SYVK) (Figure 3b), the larger fragments, *c*_14_, *c*_13_, *c*_12_, *z*_12_·, *z*_11_·, and *z*_10_· showed an overall decrease in normalized relative abundance whereas the smaller fragments *c*_11_, *c*_8,_ and *c*_7_ showed an increase in normalized relative abundance. Similar trends were observed for the doubly- and triply-phosphorylated peptides, see Supplemental Figure 2. For the doubly phosphorylated peptide (APL_p_SFRG_p_SLPKSYVK) (Supplementary Figure 2A), fragments *c*_14_, *c*_13_, *z*_13_·, and *z*_12_· show an overall decrease in normalized relative abundance with increasing electron energy, whereas *c*_12_, *c*_11_, *c*_7_, and *z*_11_· show an increase. The triply phosphorylated peptide (APL_p_SFRG_p_SLPK_p_SYVK) (Supplementary Figure 2B) shows a general decrease in normalized relative abundance for the large fragments, *c*_14_, *c*_13_, *z*_13_·, z_12_·, and z_11_·, and an increase for the fragments *c*_12_ and *c*_7_. The results support the hypothesis that deposition of increasing amounts of energy into the peptide ion enables cleavage of noncovalent bonds with associated hydrogen rearrangement, i.e., reprotonation of the phospho-group.

**Figure 4:**
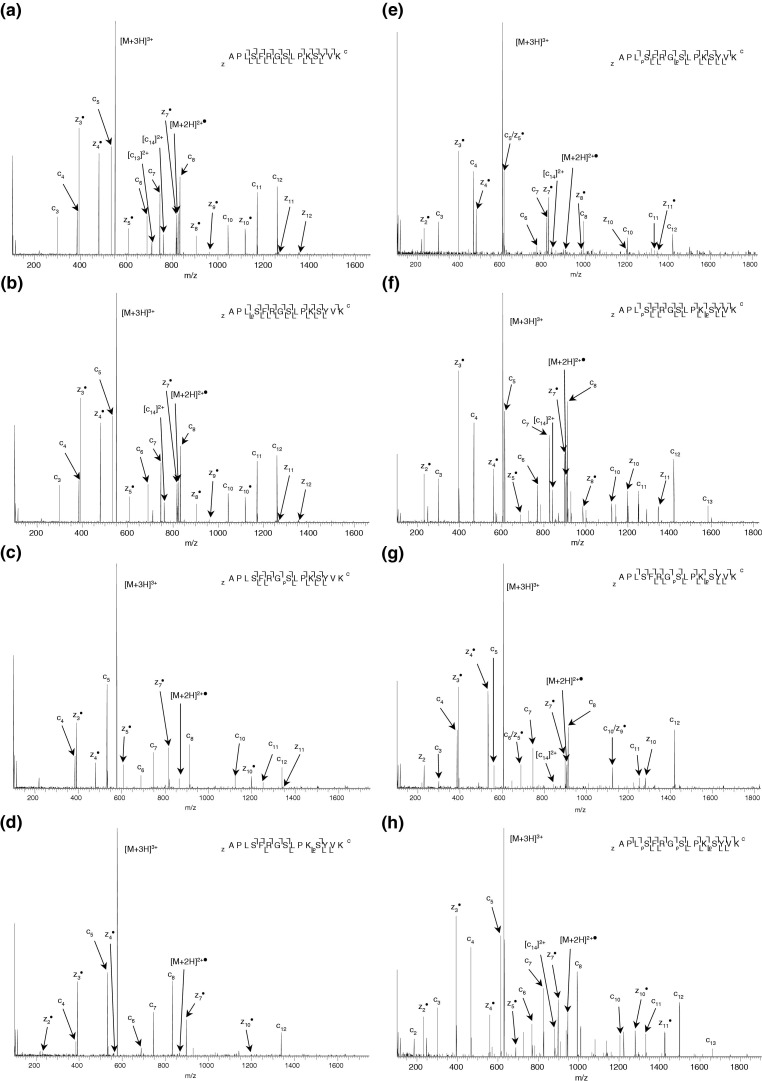
ECD mass spectra of triply-charged synthetic (APLS1FRGS2LPKS3YVK) peptide ions obtained at “standard” (−3.34 V) ECD cathode potential. (**a**) Unmodified peptide (12 scans); (**b**) Ser1 phosphopeptide (10 scans); (**c**) Ser2 phosphopeptide (12 scans); (**d**) Ser3 phosphopeptide (12 scans); (**e**) Ser1, Ser2 phosphopeptide (11 scans); (**f**) Ser1, Ser3 phosphopeptide (9 scans); (**g**) Ser2, Ser3 phosphopeptide (10 scans); (**h**) Ser1, Ser2, Ser3 phosphopeptide (9 scans).

The results above demonstrate that increasing the ECD electron energy improves sequence coverage for these phosphopeptides. Improved sequence coverage is also obtained when the triply-charged precursor ions are subjected to ECD. This finding is in agreement with that of Zubarev et al. [50], i.e., that electron capture cross section, and hence ECD efficiency, increases with the square of the ion charge. Figure 4 shows the ECD mass spectra of the triply-charged synthetic peptides acquired at the standard ECD cathode potential (−3.34 V). The ECD mass spectrum of the unmodified peptide (APLSFRGSLPKSYVK) (Figure 4a) shows cleavage of 11 out of 14 N–Cα bonds, one less than observed for the doubly-charged precursor. Two doubly-charged fragments (*c*_14_^2+^ and *c*_13_^2+^) were observed. For the singly-phosphorylated peptide (APL_p_SFRGSLPKSYVK) (Figure 4b), 10 out of 14 N–Cα bonds were cleaved, an increase of five over the doubly-charged precursor. The site of phosphorylation is unambiguously identified. Figure 4c shows the ECD mass spectrum of triply-charged peptide APLSFRG_p_SLPKSYVK. Eight of the 14 N–Cα bonds were cleaved. This is not an increase over the doubly-charged precursor; however the fragments derive from the central region of the peptide enabling identification of the site of phosphorylation. The ECD mass spectrum of the triply-charged peptide (APLSFRGSLPK_p_SYVK) (Figure 4d) shows cleavage of eight out of 14 N–Cα bonds, an increase of one cleavage over the doubly-charged precursor. The site of phosphorylation can be identified from the ECD mass spectrum of the triply-charged ions but not from the equivalent mass spectrum of the doubly-charged ions. Figure 4e shows the ECD mass spectrum for the triply-charged doubly-phosphorylated peptide APL_p_SFRG_p_SLPKSYVK. Eleven out of 14 N–Cα bonds were cleaved, five more cleavages than were observed for the doubly-charged ion. One doubly-charged fragment was observed. The ECD mass spectrum for the triply-charged peptide (APL_p_SFRGSLPK_p_SYVK) (Figure 4f) shows 11 N–Cα bonds cleaved, six more than the doubly-charged species. The *c*_14_ ion was the only doubly-charged fragment. ECD of the final doubly phosphorylated peptide (APLSFRG_p_SLPK_p_SYVK) (Figure 4g) resulted in cleavage of 11 N–Cα bonds, four more than observed for the doubly-charged ion. The ECD mass spectrum of the triply phosphorylated peptide (APL_p_SFRG_p_SLPK_p_SYVK) (Figure 4h) shows cleavage of 12 of the 14 N–Cα bonds, seven more than observed for the doubly-charged species. Only one doubly-charged fragment (*c*_14_) was observed.

Unlike the doubly-charged precursors, assigning the charging pattern based on the observed ECD fragmentation is not straightforward for the triply-charged peptide ions. For example, the charge on APL_p_SFRGSLPKSYVK could arise through deprotonation of the phospho-group and protonation of the N-terminus, the arginine, and both lysine residues, or simply through protonation of the basic residues. Contemplation of the fragmentation pattern suggests that in fact the precursors sample a number of charging patterns, each with an overall charge state of +3. Similarly, electron capture could occur at the C-terminal lysine residue or at the protonated N-terminus and the observed fragmentation suggests that both situations occur, even for the unmodified peptide. For those precursors, which contain deprotonated phospho groups, it is likely salt bridges with protonated amino acid side chains exist. However, for most N–Cα cleavages within the peptides, the resulting complementary fragments will be positively charged and will separate as a result of Coulombic repulsion.

**Figure 5:**
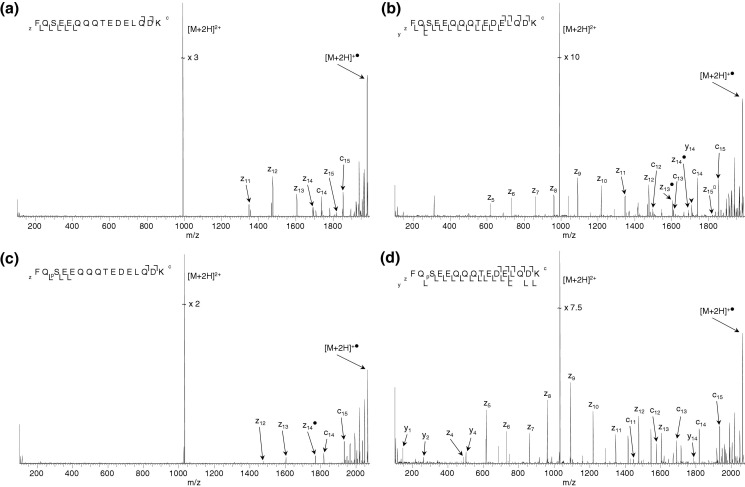
(Top) ECD mass spectra (4 scans) of doubly-charged ions of the β-casein tryptic peptide FQSEEQQQTEDELQDK obtained at ECD cathode potentials of (**a**) −3.34 V (standard) and (**b**) −9.34 V. (Bottom) ECD mass spectra (7 scans) of doubly-charged ions of FQ_p_SEEQQQTEDELQDK obtained at ECD cathode potentials of (**c**) −3.34 V (standard) and (**d**) −12.84 V.

Figure 5 shows the ECD mass spectra obtained from doubly-charged ions of the β-casein peptide (FQ(_p_)SEEQQQTEDELQDK). Figure 5a and b show the ECD mass spectra obtained from the dephosphorylated peptide at ECD cathode potentials of −3.34 V (standard) and −9.34 V (i.e., the cathode potential at which greatest sequence coverage was observed). Figure 5c and d show the equivalent ECD mass spectra obtained for the phosphopeptide. ECD of the unmodified peptide at standard cathode potential (Figure 5a) resulted in cleavage of seven out of 15 N–Cα bonds. For the phosphorylated peptide at standard ECD cathode potential (Figure 5c), cleavage of five out of the 15 N–Cα bonds was observed and the site of modification identified. As with the synthetic peptides, the increase in cathode potential results in greater peptide coverage. At a cathode potential of −9.34 V, complete sequence coverage was obtained for the unmodified peptide. At a cathode potential of −12.84 V, 13 of the 15 N–Cα bonds were cleaved in the phosphopeptide.

Unlike the phosphopeptides described above, these peptides have a single basic residue. The sequence coverage for the phosphorylated peptide is less than for the unmodified version but the difference cannot be accounted for by deprotonation of the phospho-group alone. These peptides are highly acidic. For the unmodified peptide, the fragmentation pattern at standard ECD energy (Figure 5a) suggests that Gln6 and Gln14 are protonated [51], Glu10 and Glu12 are deprotonated, and that salt bridges between the positively and negatively charged groups prevent detection of the ECD fragments. The fragmentation pattern for the phosphorylated peptide suggests that additional noncovalent interactions are present, e.g., that Glu4 is deprotonated and bound via a salt bridge to a glutamine. It is postulated that the fragmentation pattern reflects a change in conformation of the peptide as a result of phosphorylation, rather than the direct involvement of the phospho-group in any noncovalent bonding. For both peptides increasing the ECD electron energy results in a marked increase in backbone fragments, particularly *z* ions. Higher electron energy is required by the phosphopeptide to achieve maximum sequence coverage. The results suggest that deposition of additional energy cleaves any noncovalent interactions, with associated hydrogen rearrangement, enabling detection of the backbone fragments.

**Figure 6:**
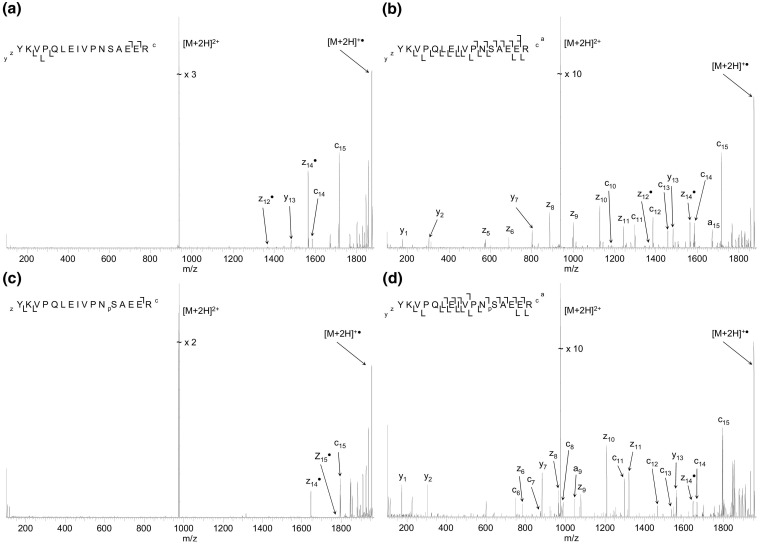
(Top) ECD mass spectra (3 scans) of doubly-charged ions of the α-S1-casein tryptic peptide YKVPQLEIVPNSAEER obtained at ECD cathode potentials (**a**) −3.34 V (standard) and (**b**) −11.84 V. (Bottom) ECD mass spectra (5 scans) of doubly-charged ions of YKVPQLEIVPN_p_SAEER obtained at cathode potentials of (**c**) −3.34 V (standard) and (**d**) −12.34 V.

Similar results were obtained following ECD of doubly-charged ions of the α-S1-casein phosphopeptide (YKVPQLEIVPN_p_SAEER) and its unmodified counterpart (see Figure 6). The mass spectrum of the unmodified peptide at the standard ECD cathode potential (Figure 6a) reveals only four N-Cα fragments (*c*_15_, *c*_14_, and *z*_14_·, *z*_12_·). The fragmentation pattern suggests that there is a noncovalent interaction between deprotonated Glu14 and protonated Gln5 [51]. There were only three fragments (*c*_15_, *z*_15_·, and *z*_14_·) observed following ECD of the phosphorylated peptide at standard cathode potential (Figure 6c). The phosphorylation site cannot be localized. The lack of fragments appears to be the result of a salt bridge between protonated Pro4 [51] and a deprotonated glutamic acid residue, in this case Glu15. (Note that we do not expect cleavage N-terminal to proline). As for the previous case, apparently the effect of phosphorylation on conformation is key rather than the involvement of the phospho-group in noncovalent bonding. The greatest sequence coverage of the unmodified peptide (Figure 6b) was obtained with an ECD cathode potential of −11.84 V; 12 out of 15 N–Cα bonds were cleaved and the *y*_7_ and *y*_13_ fragments were observed. The greatest coverage for the phosphorylated peptide (Figure 6d) was observed at a cathode potential of −12.34 V. Eleven out of fifteen N–Cα bonds were cleaved and the *y*_7_ and *y*_13_ fragments were produced. The site of phosphorylation is identified. Again, higher electron energy is required to obtain maximum sequence coverage for the phosphopeptide.

## Conclusions

We have shown that the site and frequency of phosphorylation has a marked effect on a peptide's ECD behavior. For the synthetic peptide, addition of a single phosphorylation reduced the sequence coverage from 100% (omitting proline residues) to 67% (best case) or 42% (worst case). Our results suggest that the phospho-groups exist in their deprotonated form and therefore phosphopeptide ions require additional protons to achieve a 2+ charge state. Sequence coverage of phosphopeptides can be improved by conducting ECD with higher electron energies. It is postulated that deposition of additional energy on electron capture results in cleavage of noncovalent interactions (salt bridges) between deprotonated phospho-groups and protonated amino acid side chains, accompanied in some cases by hydrogen rearrangement and reprotonation of the phospho-group. This is akin to activated ion ECD [43]. Sequence coverage can also be improved by performing ECD on the triply-charged peptide ions. This is perhaps unsurprising as ECD efficiency is known to improve with increasing charge state [50]. The presence of phosphorylation on the β-casein tryptic peptide also reduced the sequence coverage from 47% to 33%. The poor coverage for the unmodified peptide can be explained by the presence of salt bridges between deprotonated glutamic acid residues and protonated glutamine residues. The presence of the phosphorylation alters the intramolecular bonding and consequently the observed ECD fragmentation. Increasing the ECD electron energy improved sequence coverage for both peptides (100% for the unmodified peptide and 87% for the phosphopeptide). For the α-S1-casein peptide, the unmodified peptide and phosphopeptide had sequence coverages of 30% and 23% (omitting proline residues), respectively, at the standard electron energy but this increased to a maximum of 92% (unmodified) and 85% (phosphorylated) with increased electron energy. Again, this result suggests that salt bridges between deprotonated glutamic acid residues and protonated proline or glutamine prevent detection of ECD fragments at lower electron energies.

This work is important for the understanding of gas-phase fragmentation of phosphopeptides and other peptides in which relatively strong noncovalent interactions are present. The peptides studied here are either very basic (synthetic phosphopeptides) or acidic (tryptic phosphopeptides), and represent extremes of the spectrum in terms of a protein tryptic digest. Nevertheless, this work has relevance to the inclusion of ECD in global proteomic strategies. Global analyses require that optimum parameters suiting the majority of analytes to be investigated are applied. Our results suggest that LC-ECD MS/MS of an enriched phosphopeptide mixture, or an acidic strong cation exchange fraction, may require higher electron energies, or an alternative form of activation. Work in this area is ongoing.

## Supplementary data

Supplementary Figure 1 ECD mass spectra of doubly-charged ions of four synthetic peptides (APLS1FRGS2LPKS3YVK). ECD of (A) Ser1 and Ser2 phosphopeptide at cathode potentials of (left) −3.34 V and (right) −14.84 V (9 scans); (B) Ser1 and Ser3 phosphopeptide at cathode potentials of (left) −3.34 V and (right) −14.84 V (9 scans); (C) Ser2 and Ser3 phosphopeptide at cathode potentials of (left) −3.34 V and (right) −14.84 V (8 scans) and (D) Ser1, Ser2 and Ser3 phosphopeptide at cathode potentials of (left) −3.34 V and (right) −14.34 V (10 scans).

Supplementary Figure 2 Normalized relative abundance of peptide fragment ions versus ECD cathode potential for (A) the doubly modified phosphopeptide (APLpSFRGpSLPKSYVK); and (B) the triply modified phosphopeptide (APLpSFRGpSLPKpSYVK).
