# Supplementary material for: The Effect of Phosphorylation on the Electron Capture Dissociation of Peptide Ions
Source: J Am Soc Mass Spectrom. 2008 Sep;19(9):1263–74. doi: 10.1016/j.jasms.2008.05.015 (PMC2570175; doi:10.1016/j.jasms.2008.05.015)
Supplement: Supplementary Figure 1 — ECD mass spectra of doubly-charged ions of four synthetic peptides (APLS1FRGS2LPKS3YVK). ECD of (A) Ser1 and Ser2 phosphopeptide at cathode potentials of (left) −3.34 V and (right) −14.84 V (9 scans); (B) Ser1 and Ser3 phosphopeptide at cathode potentials of (left) −3.34 V and (right) −14.84 V (9 scans); (C) Ser2 and Ser3 phosphopeptide at cathode potentials of (left) −3.34 V and (right) −14.84 V (8 scans) and (D) Ser1, Ser2 and Ser3 phosphopeptide at cathode potentials of (left) −3.34 V and (right) −14.34 V (10 scans). [file mmc1.ppt]

## Slide 1
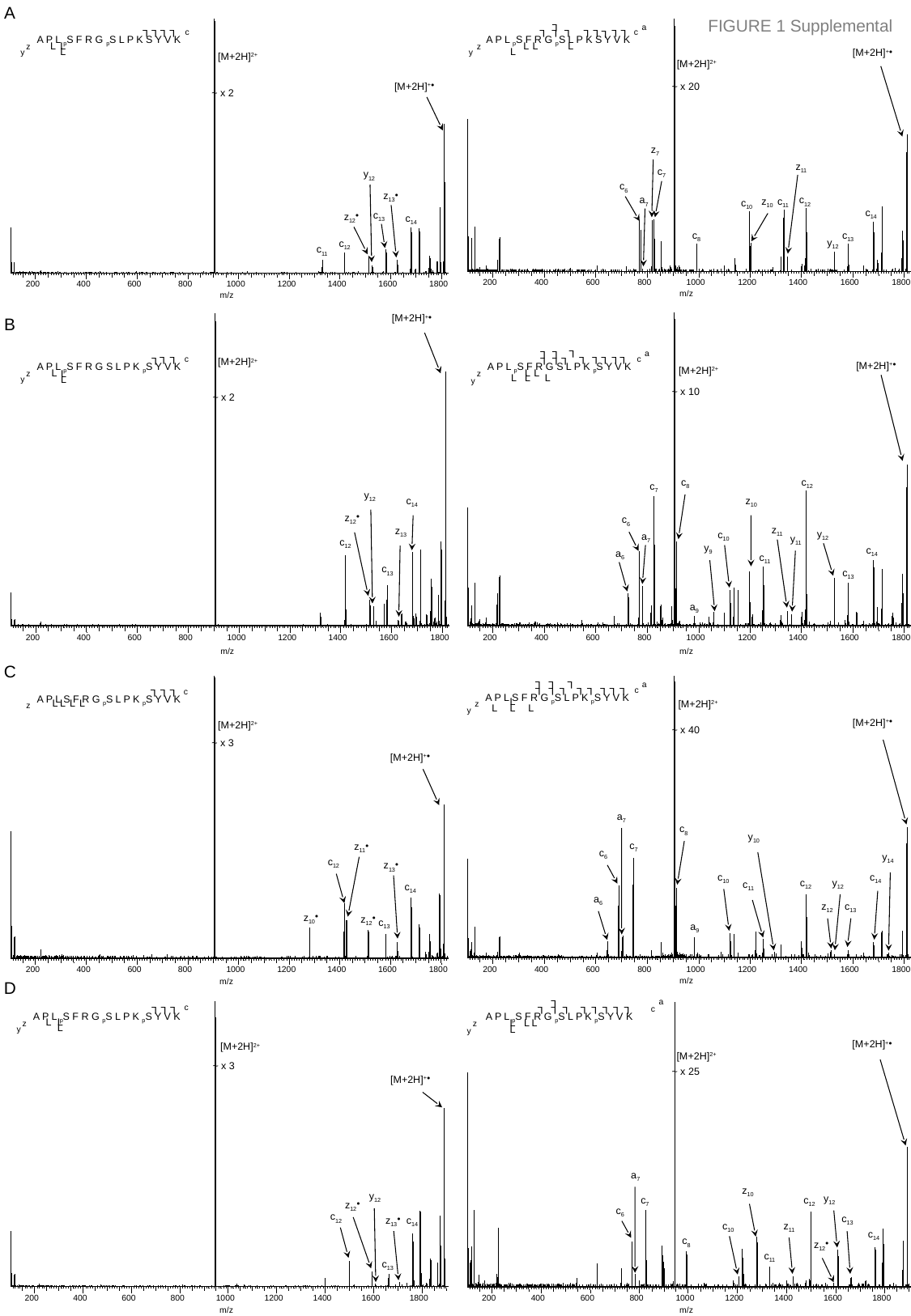

A
FIGURE 1 Supplemental
200
400
600
800
1000
1200
1400
1600
1800
m/z
[M+2H]2+
[M+2H]+●
y12
z13●
c13
z12●
c14
c12
c11
200
400
600
800
1000
1200
1400
1600
1800
m/z
a
L
c
c
L
L
L
L
L
L
L
L
L
L
L
L
A P L pS F R G pS L P K S Y V K
A P L pS F R G pS L P K S Y V K
L
L
L
L
L
z
z
L
L
[M+2H]+●
y
y
[M+2H]2+
~ x 20
~ x 2
z7
z11
c7
c6
a7
c12
z10
c11
c10
c14
c8
c13
y12
[M+2H]+●
200
400
600
800
1000
1200
1400
1600
1800
m/z
[M+2H]2+
y12
c14
z12●
z13
c12
c13
B
200
400
600
800
1000
1200
1400
1600
1800
m/z
a
L
L
L
c
c
L
L
L
L
L
L
L
L
L
L
L
[M+2H]+●
A P L pS F R G S L P K pS Y V K
A P L pS F R G S L P K pS Y V K
[M+2H]2+
L
L
L
L
z
z
L
L
L
L
y
y
~ x 10
~ x 2
c8
c12
c7
z10
c6
z11
y12
c10
a7
y11
y9
c14
a6
c11
c13
a9
C
200
400
600
800
1000
1200
1400
1600
1800
m/z
[M+2H]2+
[M+2H]+●
z11●
c12
z13●
c14
z10●
z12●
c13
[M+2H]2+
[M+2H]+●
a7
c8
y10
c7
c6
y14
c10
c14
c12
y12
c11
a6
z12
c13
a9
200
400
600
800
1000
1200
1400
1600
1800
m/z
a
L
L
L
c
L
L
L
L
L
L
L
L
c
L
L
L
A P L S F R G pS L P K pS Y V K
A P L S F R G pS L P K pS Y V K
L
L
L
L
L
z
z
L
L
L
y
~ x 40
~ x 3
D
a
L
c
200
400
600
800
1000
1200
1400
1600
1800
m/z
200
400
600
800
1000
1200
1400
1600
1800
m/z
c
L
L
L
L
L
L
L
L
L
L
L
A P L pS F R G pS L P K pS Y V K
A P L pS F R G pS L P K pS Y V K
L
L
L
L
L
z
z
L
L
y
y
[M+2H]+●
[M+2H]2+
[M+2H]2+
~ x 3
~ x 25
[M+2H]+●
a7
z10
y12
y12
c7
c12
z12●
c6
c12
c13
z13●
c14
c10
z11
c14
c8
z12●
c11
c13
