# Supplementary figures and images for: The Effect of Phosphorylation on the Electron Capture Dissociation of Peptide Ions
Source: J Am Soc Mass Spectrom. 2008 Sep;19(9):1263–74. doi: 10.1016/j.jasms.2008.05.015 (PMC2570175; doi:10.1016/j.jasms.2008.05.015)

## Slide 1
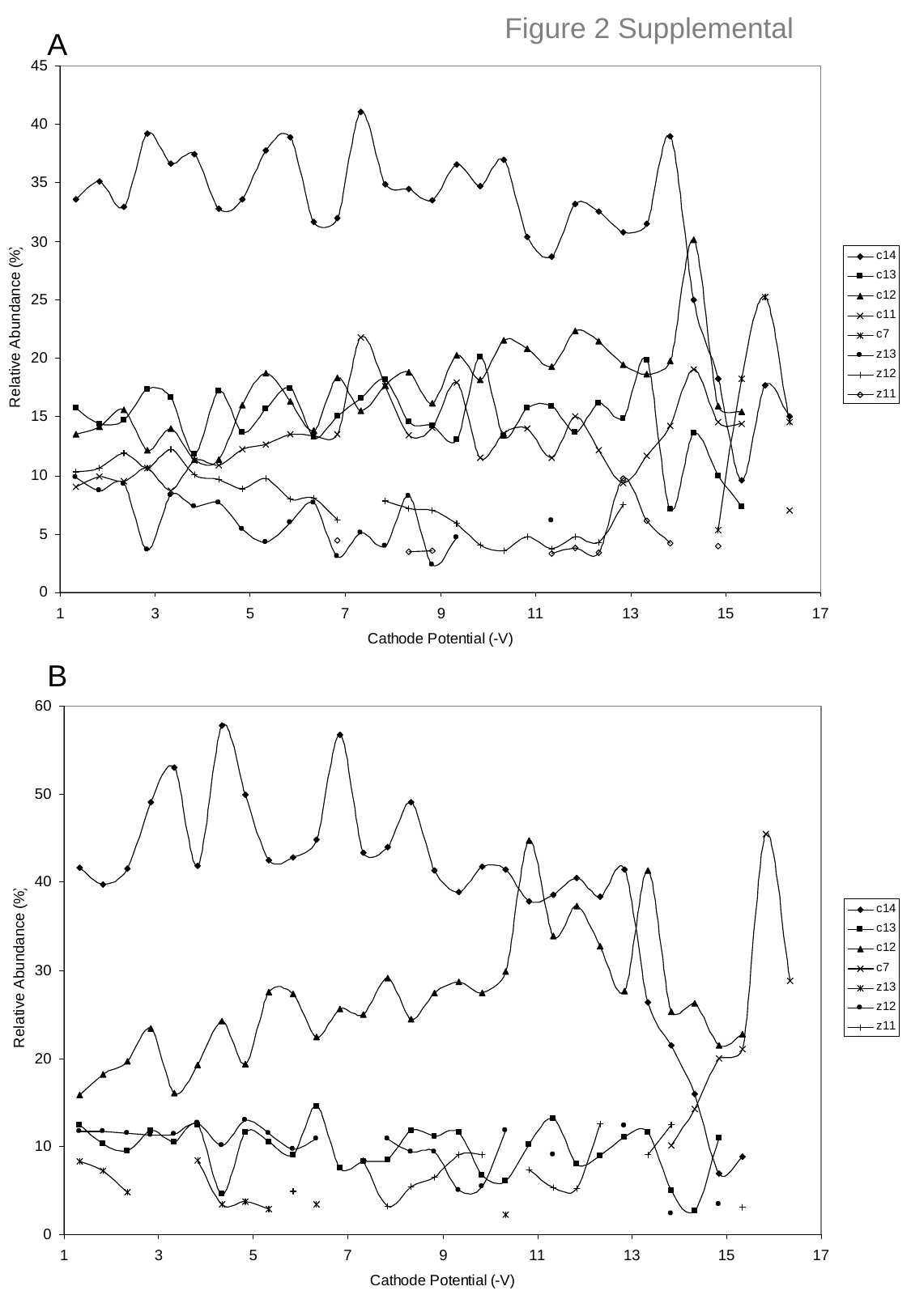

Figure 2 Supplemental
A
B

Supplement: Supplementary Figure 2 — Normalized relative abundance of peptide fragment ions versus ECD cathode potential for (A) the doubly modified phosphopeptide (APLpSFRGpSLPKSYVK); and (B) the triply modified phosphopeptide (APLpSFRGpSLPKpSYVK). [file mmc2.ppt]
